# Transcriptional Changes Associated with Amyoplasia

**DOI:** 10.3390/ijms26010124

**Published:** 2024-12-26

**Authors:** Artem E. Komissarov, Olga E. Agranovich, Ianina A. Kuchinskaia, Irina V. Tkacheva, Olga I. Bolshakova, Evgenia M. Latypova, Sergey F. Batkin, Svetlana V. Sarantseva

**Affiliations:** 1 Petersburg Nuclear Physics Institute Named by B.P. Konstantinov of National Research Centre “Kurchatov Institute”, Gatchina 188300, Russia; komissarov_ae@pnpi.nrcki.ru (A.E.K.); kuchinskaya_yaa@pnpi.nrcki.ru (I.A.K.); iritka4eva@gmail.com (I.V.T.); bolshakova_oi@pnpi.nrcki.ru (O.I.B.); latypova_em@pnpi.nrcki.ru (E.M.L.); 2 H. Turner National Medical Research Center for Children’s Orthopedics and Trauma Surgery, Saint Petersburg 196603, Russia; olga_agranovich@yahoo.com (O.E.A.); sergey-batkin@mail.ru (S.F.B.)

**Keywords:** arthrogryposis multiplex congenita (AMC), amyoplasia, transcriptomic analysis, contractures, mitochondria

## Abstract

Arthrogryposis, which represents a group of congenital disorders, includes various forms. One such form is amyoplasia, which most commonly presents in a sporadic form in addition to distal forms, among which hereditary cases may occur. This condition is characterized by limited joint mobility and muscle weakness, leading to limb deformities and various clinical manifestations. At present, the pathogenesis of this disease is not clearly understood, and its diagnosis is often complicated due to significant phenotypic diversity, which can result in delayed detection and, consequently, limited options for symptomatic treatment. In this study, a transcriptomic analysis of the affected muscles from patients diagnosed with amyoplasia was performed, and more than 2000 differentially expressed genes (DEGs) were identified. A functional analysis revealed disrupted biological processes, such as vacuole organization, cellular and aerobic respiration, regulation of mitochondrion organization, cellular adhesion, ATP synthesis, and others. The search for key nodes (hubs) in protein–protein interaction networks allowed for the identification of genes involved in mitochondrial processes.

## 1. Introduction

Arthrogryposis multiplex congenita (AMC) describes a group of heterogeneous conditions characterized by multiple non-progressive joint contractures in two or more are-as, with or without muscle weakness. These contractures vary in distribution and severity [1]. AMC causes articular stiffness, limiting the range of motion and negatively impacting activities of daily living—such as ambulation, feeding, or toileting—and social participation, such as the ability to work. AMC is usually non-progressive and often gradually improves with proper management [1,2]. There are two major types of AMC: the most common one is amyoplasia, characterized by symmetrical contractures, usually with internally rotated and adducted shoulders, overstretched elbows, flexed wrists, distal flexion contractures in interphalangeal joints, adducted thumbs, hip joint, flexed or overstretched knee, and clubfoot [3]. The second major form is distal arthrogryposis, a group of genetic diseases, that affects the distal parts of the limbs, hands, and feet, with limited damage to the proximal joints and variable expressivity [3,4]. The incidence of AMC ranges from 1:3000 to 1:5100 live births [4]. More than 400 specific conditions leading to the development of arthrogryposis have been described [5,6]. The main prerequisite for the occurrence of arthrogryposis is a decrease in fetal mobility during intrauterine development, which can be caused by many reasons, such as various pathologies of the mother’s pregnancy or defects in the nervous system of the fetus [7,8,9,10]; muscle disorders, such as congenital muscular dystrophies and myopathies, intrauterine myositis, and mitochondrial disorders [11,12]; as well as the impacts of various harmful factors of physical and chemical nature on the embryo in the early stages of its formation [13].

In recent years, with the development of the availability of exome and genomic sequencing, mutations have been identified in the genes of proteins that control the structure and function of motor neurons, neuromuscular junctions and skeletal muscles, which lead to the development of distal arthrogryposis [14,15,16,17,18], accounting for approximately 65% of all cases of AMC [19].

Amyoplasia (or classic arthrogryposis) is characterized by the most severe disorders and occurs with a frequency of 1/10,000 newborns [20]. Most cases of amyoplasia are sporadic and may, as suggested, have an epigenetic nature [21]; however, for the most severe cases, the role of hereditary factors has also been shown [22].

To gain deeper insight into the pathogenesis of arthrogryposis in humans, we studied the transcriptome of muscle samples from 11 AMC and age- and sex-matched controls. We used Gene Ontology (GO) pathway analyses to assess which functional pathways the identified genes were involved in. We then applied protein–protein interaction (PPI) network analysis to identify the most important hub genes mediating the effects in patient tissues.

## 2. Results

### 2.1. Transcriptomic Analysis of Amyoplasia Muscle Samples

**Figure 1:**
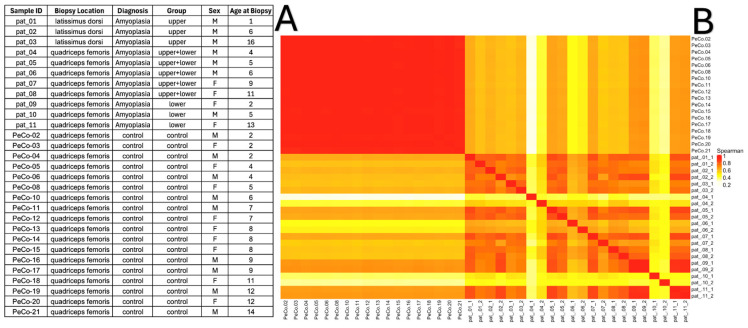
(**A**) List of muscle biopsy samples subjected to RNA-seq; and (**B**) heatmap displaying the Spearman correlations between samples.

To gain insight into the extent of transcriptomic dysregulation in amyoplasia, we performed total RNA-seq on 11 skeletal muscle samples collected from amyoplasia individuals. Furthermore, 18 age- and sex-matched muscle samples from healthy individuals from the open-source GEO (accession number GSE201255) [23] were used as controls. Figure 1A and the Materials and Methods section detail the main characteristics of the patients and healthy controls. We also examined the correlations between the samples. Figure 1B shows that the control samples were strongly correlated with each other while, among the experimental samples, the correlation values were within the range of 0.7–0.8. For two samples (pat_4 and pat_10), the correlations were lower and they fell out of the overall picture; however, we decided not to exclude these samples from the analysis.

**Figure 2:**
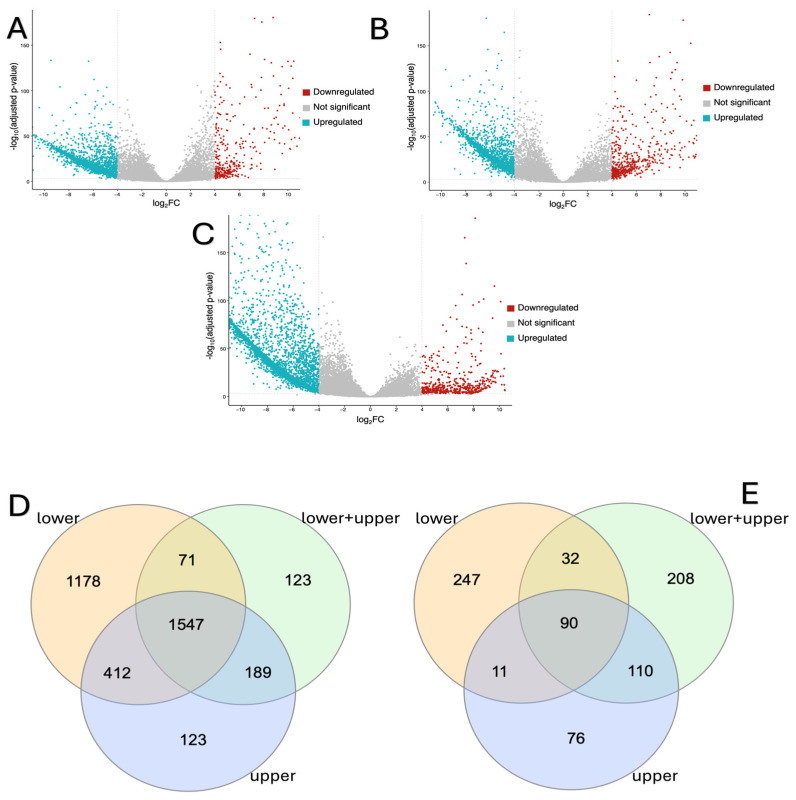
RNA-seq analysis showing differential gene expression in amyoplasia muscle and control muscle. Volcano plots showing −log (adjusted *p*-value) vs. log2 (fold change) for “lower” (**A**), “upper+lower” (**B**), and “upper” (**C**) groups. Dashed vertical lines mark log2 (fold change) > |4|. Dashed horizontal line marks adjusted *p*-value < 0.001. Blue dots represent downregulated genes and red dots represent upregulated genes. Venn plots illustrating the intersection of (**D**) downregulated DEGs and (**E**) upregulated DEGs between “lower”, “lower+upper”, and “upper” groups.

We found that 3208 genes were downregulated and 380 genes were upregulated in the “lower” group (Figure 2A; Supplementary Material S1), 1930 genes were downregulated and 440 genes were upreg-ulated in the “upper+lower” group, when compared with the controls (Figure 2B; Supplementary Material S2) and 2271 genes were downregulated and 287 genes were upregulated in the “upper” group (Figure 2C; Supplementary Material S3). Notably, the highest number of differentially expressed genes was observed in the “lower” group. This group also had a higher number of downregulated genes. Volcano plots illustrate the expression patterns of differentially expressed genes (DEGs) between AMC samples and the control group (Figure 2A–C).

In the “lower” and “upper” groups, the genes with the greatest degree of downregulation were *MYH2* (log2(fc) = −15), *MYH1* (log2(fc) = −15)*, RN7SL1* (log2(fc) = −15)*, MYOT* (log2(fc) = −12) and *COQ8A* (log2(fc) = −12), while those in the “upper+lower” group were *MYH2* (log2(fc) = −15)*, RN7SL1* (log2(fc) = −14)*, MYH1* (log2(fc) = −14)*, TTN* (log2(fc) = −11) and *PDK4* (log2(fc) = −14). In all studied groups, the expression of the *TMOD4* (log2(fc) = 17 for “upper”, log2(fc) = 16 for “lower+upper” and “lower” groups) and *MIR4300HG* (log2(fc) = 14 for “upper”, log2(fc) = 13 for “lower+upper” and log2(fc) = 12 for “lower” group) genes was the most increased. In addition, in the “lower” group, genes with the greatest degree of upregulation included *LINCMD* (log2(fc) = 11), *ARL14EPL* (log2(fc) = 11) and *RPS18P12* (log2(fc) = 11); those in the “upper” group were *RPL26P36* (log2(fc) = 12), *RMRP* (log2(fc) = 11) and *LINC00845* (log2(fc) = 11); and those in the “upper+lower” group were *CNTNAP5* (log2(fc) = 13)*, NRXN1-DT* (log2(fc) = 12) and *LINC01564* (log2(fc) = 11).

Finally, comparison of the DEGs between study groups revealed that 1547 genes were downregulated and 173 genes were upregulated in all three groups (Figure 2D,E; Supplementary Material S4).

### 2.2. Identification of Key Biological Pathways

Next, to understand the roles of the genes and their interactions in various biological processes when comparing patients from different amyoplasia groups, we performed Gene Ontology (GO) enrichment analysis using ClusterProfiler. GO classifies the characteristics of genes and gene products into three domains: biological processes, molecular functions and cellular components.

**Figure 3:**
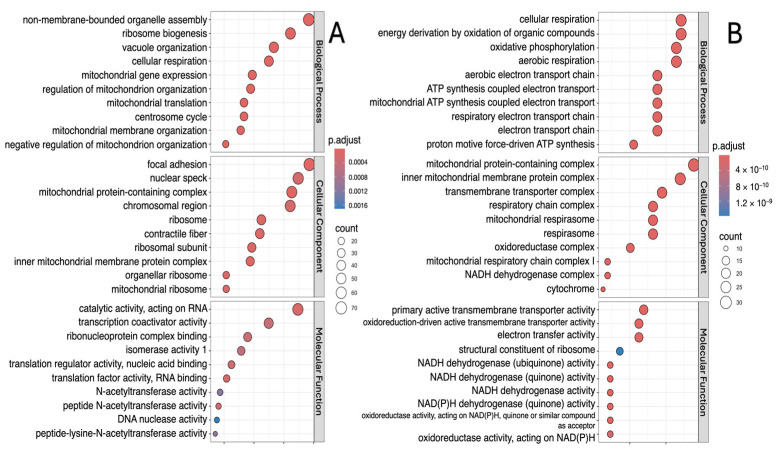
Gene Ontology function and pathway enrichment analysis of downregulated (**A**) and upregulated (**B**) DEGs in the “lower” group. Dotplots for each of the GO analysis categories (biological processes, molecular function and cellular component) are presented.

**Figure 4:**
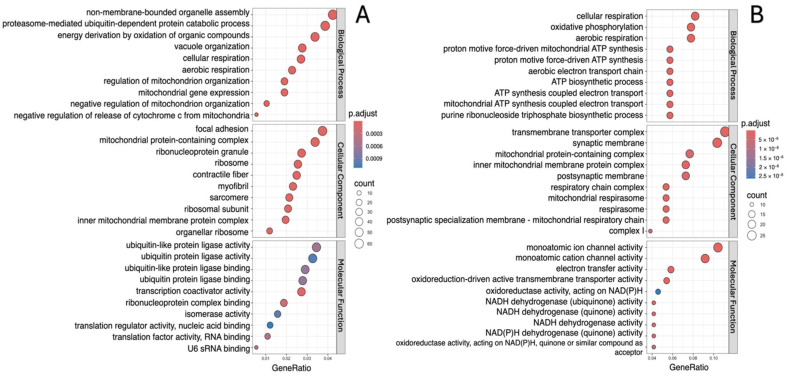
Gene Ontology function and pathway enrichment analysis of downregulated (**A**) and upregulated (**B**) DEGs in the “lower+upper” group. Dotplots for each of the GO analysis categories (biological processes, molecular function and cellular component) are presented.

**Figure 5:**
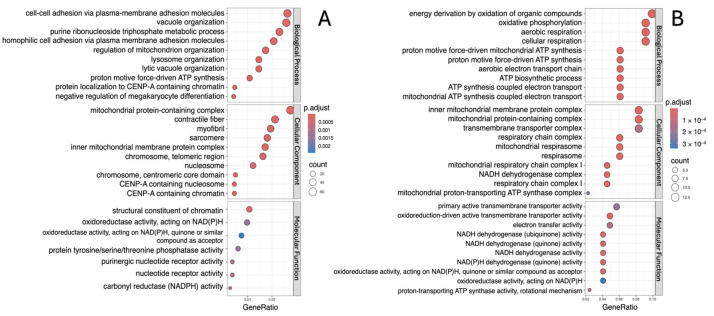
Gene Ontology function and pathway enrichment analysis of downregulated (**A**) and upregulated (**B**) DEGs in the “upper” group. Dotplots for each of the GO analysis categories (biological processes, molecular function and cellular component) are presented.

The analysis showed that, in all study groups, the downregulated DEGs are involved in biological processes such as vacuole organization, cellular and aerobic respiration, and regulation of mitochondrion organization (Figure 3A, Figure 4A and Figure 5A). Moreover, in addition to those indicated above, processes associated with cellular adhesion were identified for the “lower” group (Figure 3A). Meanwhile, upregulated DEGs were characterized by involvement in the processes of cellular respiration, oxidative phosphorylation, aerobic respiration and ATP synthesis (Figure 3B, Figure 4B and Figure 5B).

The molecular functions associated with downregulated DEGs were mainly structural constituent of chromatin, biosynthesis and activity of NAD(P)H (“lower” group); ubiquitin-like protein ligase activity and ubiquitin protein ligase activity (“upper+lower” group); and catalytic activity, acting on RNA, transcription coactivator activity and ribonucleoprotein complex binding (“upper” group). In all study groups, the molecular functions associated with upregulated DEGs were mainly transmembrane transporter activity, oxidoreduction-driven active and NADH dehydrogenase activity (Figure 3, Figure 4 and Figure 5).

The most enriched cellular component terms for downregulated DEGs were mitochondrial protein-containing complex, contractile fiber, inner mitochondrial membrane protein complex, focal adhesion, myofibril and sarcomere. On the other hand, the most enriched cellular component terms for upregulated DEGs were mainly mitochondrial protein-containing complex, inner mitochondrial membrane protein complex, transmembrane transporter complex, respiratory chain complex, and mitochondrial respirasome (Figure 3, Figure 4 and Figure 5).

### 2.3. Protein–Protein Interaction Network Construction and Identification of the Hub Genes

**Figure 6:**
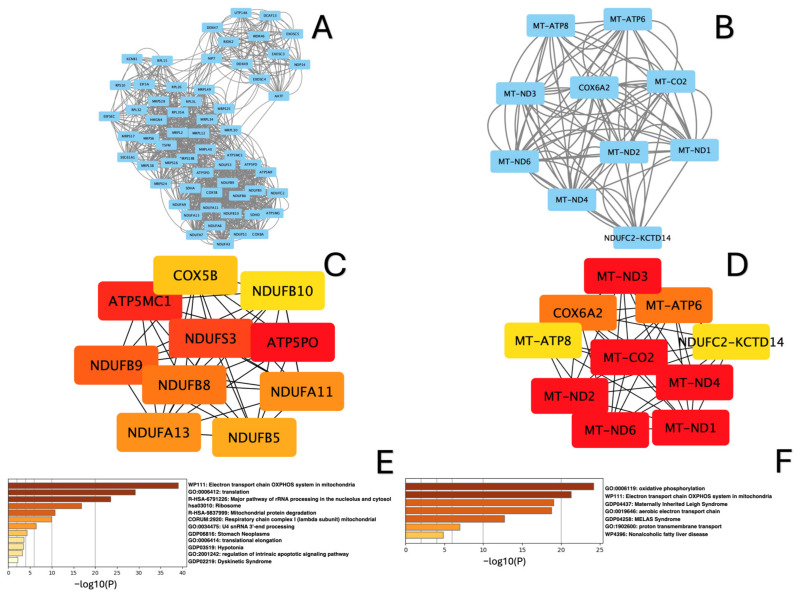
Analysis of PPI networks for “lower” group sample: (**A**) MCODE-clustered subnetwork for downregulated DEGs; (**B**) MCODE-clustered subnetwork for upregulated DEGs. Hub genes identified by cytoHubba. (**C**) Hub genes of the PPI network for downregulated DEGs; (**D**) Hub genes of the PPI network for upregulated DEGs. Enrichment analysis of MCODE-clustered subnetwork by Metascape. (**E**) Enrichment analysis of downregulated DEGs; (**F**) Enrichment analysis of upregulated DEGs.

**Figure 7:**
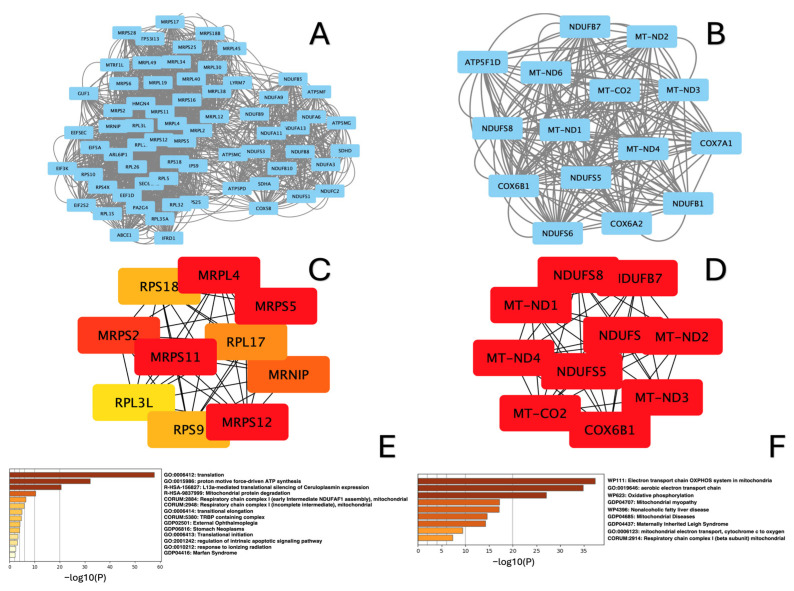
Analysis of PPI networks for “upper+lower” group sample. (**A**) MCODE-clustered subnetwork for downregulated DEGs; (**B**) MCODE-clustered subnetwork for upregulated DEGs. Hub genes identified by cytoHubba. (**C**) Hub genes of the PPI network for downregulated DEGs; (**D**) Hub genes of the PPI network for upregulated DEGs. Enrichment analysis of MCODE-clustered subnetwork by Metascape. (**E**) Enrichment analysis of downregulated DEGs; (**F**) Enrichment analysis of upregulated DEGs.

**Figure 8:**
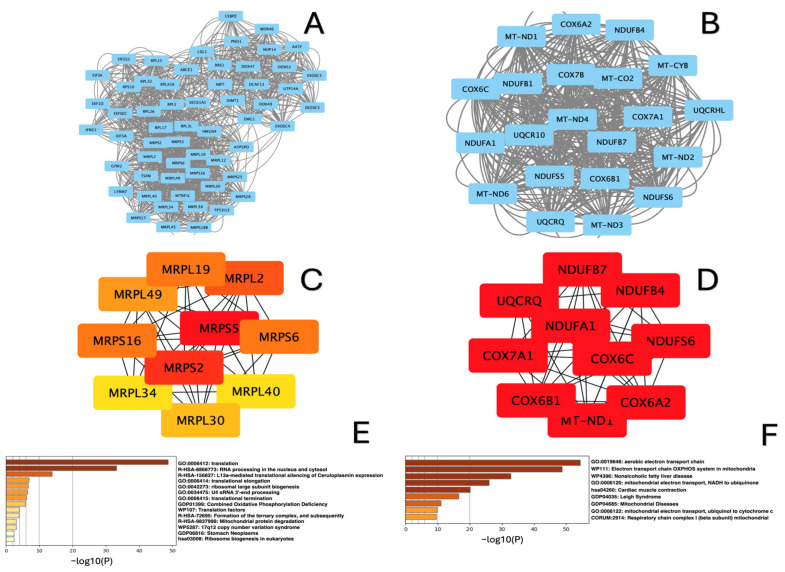
Analysis of PPI networks for “upper” group sample. (**A**) MCODE-clustered subnetwork for downregulated DEGs; (**B**) MCODE-clustered subnetwork for upregulated DEGs. Hub genes identified by cytoHubba. (**C**) Hub genes of the PPI network for downregulated DEGs; (**D**) Hub genes of the PPI network for upregulated DEGs. Enrichment analysis of MCODE-clustered subnetwork by Metascape. (**E**) Enrichment analysis of downregulated DEGs; (**F**) Enrichment analysis of upregulated DEGs.

Protein–protein interaction networks (PPI) for up- and downregulated DEGs in each study group were generated, as shown in Figure 6A,B, Figure 7A,B and Figure 8A,B. The top genes, ranked by connectivity, were screened and identified as hub genes in the context of amyoplasia. As a result, in the “lower” group, ten hub genes for the downregulated DEGs (*ATP5PO, ATP5MC1*, *NDUFS3 NDUFB9*, *NDUFB8*, *NDUFA11*, *NDUFA13*, *NDUFB5*, *NDUFB10, Cox5B*) and ten hub genes for the upregulated DEGs (*MT-ND1, MT-ND2, MT-ND3, MT-ND4*, *MT-CO2*, *MT-ND6*, *MT-ATP8*, *MT-ATP6*, *Cox6A2*, *NDUFC2-RCTD14*) were identified (Figure 6B,C). In the “upper+lower” group, the five hub genes in the network of downregulated DEGs were *MRPL4*, *MRPS5*, *MRPS2*, *MRPS1*, *RPS18, RPL17*, *RPL34, RPS9, MRNIP* and *MRPS12,* while the ten hub genes in the network of upregulated DEGs were *MT-ND1*, *MT-ND2*, *MT-ND3*, *MT-ND4*, *MT-CO2*, *NDUFS8*, *NDUFB7*, *NDUFB1*, *NDUFS6* and *NDUFS5* (Figure 7B,C). In the “upper” group, the ten hub genes in the network of downregulated DEGs included *MRPS2*, *MRPS5* and *MRPL2*, *MRPL19*, *MRPL49, MRPL34*, *MRPL30*, *MRPL40*, *MRPS6*, *MRPS16*, while the ten hub genes in the network of upregulated DEGs included *NDUFB7*, *NDUFB4*, *NDUFA1*, *NDUFS6*, *MT-ND4*, *COX7A1, COX6C*, *COX6B1*, *COX6A2* and *UQCRQ* (Figure 8B,C).

Based on the analysis using Metascape, we found that the hub genes in all three groups were mainly enriched in terms such as translation, rRNA processing in the nucleolus and cytosol, and mitochondrial protein degradation for the downregulated DEGs (Figure 6C, Figure 7C and Figure 8C), and electron transport chain OXPXOS system in mitochondria aerobic electron transport chain and oxidative phosphorylation for the upregulated DEGs (Figure 6D, Figure 7D and Figure 8D).

## 3. Discussion

AMC cases vary widely in their genetic origin, pathophysiology and clinical presentation. Given the multifactorial nature of this disease, patients with AMC require a multidisciplinary approach for proper diagnosis, treatment, and follow-up [3]. The fundamental etiology and pathophysiological mechanisms of the disease remain poorly understood which, in turn, hinders the development of effective treatments and the search for potential markers of the disease.

In this study, for the first time (to the best of our knowledge), we performed a comprehensive gene expression profile study in muscle samples from patients with amyoplasia and healthy age- and sex-matched controls. Our results showed that differentially expressed genes were predominantly enriched in pathways related to vacuole organization, cellular and aerobic respiration, regulation of mitochondrion organization, cellular adhesion and ATP synthesis.

It should be noted that, among the genes with increased expression in all three studied groups, the *TMOD4* gene was present. This gene encodes the Tropomodulin 4 protein, which is part of the tropomodulin protein family. These proteins play an important role in regulating the structure and stability of actin filaments in cells. Tmod4, in particular, is involved in maintaining the length of actin filaments, preventing their addition or removal at one of the ends. It is interesting to note the fact that, with *TMOD4* overexpression in muscle tissue, a patient may experience limb girdle muscular dystrophy 1B [24]. It has also been shown that increased *Tmod4* expression promotes adipogenesis through increasing the levels of adipogenic factors such as C/EBPα, which leads to the differentiation of fat cell precursors into mature adipocytes. At the same time, Tmod4 can inhibit myogenesis through suppressing the expression of myogenic factors such as MyoD [25]. In congenital multiple contractures, muscle tissue can be replaced by fat [26]. Presumably, this phenotype may be associated with the fact that there is hyperexpression of the *TMOD4* gene, which shifts the balance towards the development of adipose tissue.

To screen the key genes of amyoplasia, we used the STRING database to construct a PPI DEG network and identified downregulated DEGs. Surprisingly, all of the associated proteins are associated with different aspects of mitochondrial function. The identified downregulated hub genes encode mitochondrial ribosomal proteins (MRPs). MRPs are encoded by nuclear genes and synthesized by the cytoplasm 80S ribosomes, after specific targeting, sorting and transport to mitochondria, followed by assembling into mitochondrial ribosome small and large subunits [27]. The mitochondrial ribosome, composed of approximately 80 MRPs [28], conducts mitochondrial translation to produce essential electron transport chain complex protein subunits encoded by mitochondrial DNA [28].

MRPs have been reported to participate in many cellular processes, such as cell proliferation, apoptosis and cell cycle, and the abnormal expression of MRPs and their encoding genes is closely associated with a variety of pathological conditions [29].

All identified upregulated hub genes are directly involved in oxidative phosphorylation and adenosine triphosphate (ATP) production. Mitochondria play an important role in regulating the life and death of eukaryotic cells, providing energy in the form ATP through a series of oxidative phosphorylation (OXPHOS) processes [30]. Mitochondrial NADH:ubiquinone oxidoreductase (Complex I) is a complex of the mitochondrial respiratory chain involved in the OXPHOS pathway and the generation of ATP. Complex I consists of 45 subunits, 7 of which are encoded by mtDNA called mitochondrial-encoded NADH dehydrogenases (MT-NDs), including *MT-ND1, MT-ND2, MT-Nd3, MT-ND4, MT-ND4L, MT-NDS* and *MT-ND6* [31]. Consequently, dysfunction of MT-ND genes can lead to mitochondrial anterior chain dysfunction and decreased ATP production. It was also noted that other upregulated genes are associated with Complex I (*NDUFS6, NDUFS8, NDUFB7,NDUFB1, NDUFS5*), Complex III (*UQCRQ*) and Complex IV (*MT-C02, COX7A1, COX6C, COX6B1, COX6A2*) in the respiratory chain.

To date, there is no data indicating a direct link between the hub genes we identified and amyoplasia. However, Wilnai et al. have presented a clinical description of a patient with amyoplasia and mitochondrial respiratory chain complex IV deficiency caused by SURF1 deficiency. The authors speculated that, due to the similarity in the distribution of mitochondrial DNA abnormalities and amyoplasia, the two conditions may co-occur by chance. However, given the central role of mitochondria in energy production and neuromuscular function, it remains possible that the association is, indeed, causal [32]. Complex I Deficiency in skeletal muscle has been described in a neonate with severe AMC [33]. Furthermore, a mitochondrial MELAS mutation was described in a patient with distal arthrogryposis. However, the authors were unable to determine whether the occurrence of DA and MELAS in the same patient was a coincidence or whether arthrogryposis was secondary to the neuromuscular manifestations of MELAS [34].

The need for a deeper understanding of the molecular and cellular mechanisms of arthrogryposis requires animal studies. It should be noted that arthrogryposis in both humans and animals has common features and similar mechanisms of development. In animals, contractures may be caused by various factors: toxic chemicals or drugs, mechanical immobilization, viruses, gene mutations [35]. To date, there are no known animal models of amyoplasia. At the same time, intensive studies of distal arthrogryposis are being conducted in cattle, *Drosophila melanogaster*, zebrafish and mice [36,37,38]. It should be noted that mitochondrial pathology was not directly studied in these studies, but a number of authors note changes in ATPase activity [39,40,41].

Previously, Hall J.G. and Kiefer J. identified genes associated with arthrogryposis using Gene Ontology Analysis [13,42]. Their analysis was based on publications presented in Medline, PubMed, and OMIM. The selection criteria were mutation identification, description of clinical features of patients, clinical description of multiple congenital contractures. At the time of publication of the results in 2019, 402 genes had been identified which, when mutated, are associated with arthrogryposis. These genes were reported to be involved in synaptic transmission, muscle development and differentiation, central nervous system development and differentiation, and other processes [42]. This list of genes does not include the hub genes identified in this study, which may be explained by the selection method, with one of the important criteria of which being the presence of an identified mutation in patients. Thus, our study showed that dysfunction of mitochondrial respiratory chain and mitochondrial protein synthesis may be involved and play a central role in the development of amyoplasia.

However, it is important to note that our study has limitations such as a small sample size, the use of data obtained from different sources, and a reliance on bioinformatic analysis. Further studies on the genetic mechanisms responsible for AMC are needed to confirm our results.

In conclusion, our study adds to the knowledge regarding the transcriptomic landscape of AMC and its most severe form, amyoplasia. The identified differential expression of key genes and pathways provides important insights into the molecular mechanisms underlying the pathology of AMC. We hope that the presented results enhance our understanding of the disease’s pathogenesis and facilitate the development of more effective diagnostic and therapeutic strategies and biomarkers for this pathology.

## 4. Materials and Methods

### 4.1. Patient Cohort and Muscle Samples

The research study was approved by the Local Ethics Committee of H. Turner National Medical Research Center for Children’s Orthopedics and Trauma Surgery of the Ministry of Health of the Russian Federation No. 19-3 of 9 December 2019.

Transcriptome analysis was performed in 11 patients diagnosed with amyoplasia followed up in the arthrogryposis unit of the H. Turner National Medical Research Center for Children’s Orthopedics and Trauma Surgery. The limb findings in amyoplasia congenita were usually symmetric, mostly involving all four extremities. However, some patients had only the lower or upper extremities affected. Patients with AMC affecting only the upper extremities presented typical positioning, in which the shoulders were internally rotated, the elbows were extended, and the wrists and hands were flexed. Patients with AMC affecting only the lower limbs presented involved contractures around the hips (flexion, abduction and external rotation contractures), as well as hip dislocation. The knees were flexed or hyperextended and the feet were in an equinovarus position. The muscle mass of the limbs was diminished and replaced by fibrous tissue. Of the 11 patients, 3 patients had lesions of the upper limbs (hereinafter referred to as the “upper” group), 3 patients had lesions of the lower limbs (hereinafter referred to as the “lower” group) and 5 children had symmetric involvement of the upper and lower limbs (upper+lower group). In the upper extremity, the shoulders were internally rotated, the elbows were extended, the wrists were flexed and ulnarly deviated, the fingers were stiff, and the thumbs were positioned in the palm. In the lower limbs, the hips had flexion, abduction and external rotation contractures, the knees were flexed, and the feet had severe equinovarus contractures.

Muscle samples were collected during elective corrective surgical procedures. Samples were snap-frozen in liquid nitrogen and stored at −80 °C until processing. Skeletal muscle transcriptome sequencing samples from the open-source GEO (accession number GSE201255) were used as controls. These samples have been described in the study by Hale et al. [23].

### 4.2. Isolation of Total RNA from Muscle Tissue

To isolate total cellular RNA, fragments of muscle tissue were washed in phosphate-buffered saline via centrifugation at 1200× *g* for 4 min at 4 °C, and this procedure was repeated three times. Subsequently, the tissue was homogenized via repeated freezing in liquid nitrogen followed by grinding with a pestle. Total RNA was extracted using TRIzol reagent (Thermo Fisher Scientific, Carlsbad, CA, USA).

### 4.3. Purification of Total RNA from DNA Contaminants

To purify total RNA from genomic DNA, 1500 ng of the total RNA solution obtained was transferred to a separate tube and treated with DNase I, according to the standard protocol.

### 4.4. Depletion of RNA

To deplete ribosomal RNA, the Library Preparation VAHTS mRNA Capture Beads kit (Vazyme, Nanjing, China) was utilized, in accordance with the standard protocol. The efficacy of rRNA purification was subsequently assessed via real-time PCR.

### 4.5. Determination of Total RNA Concentration

The concentration of the resultant total RNA was quantified using a fluorometer (Qubit 4 Fluorometer, Thermo Fisher Scientific, Waltham, MA, USA) with a Qubit RNA HS Kit (Thermo Fisher Scientific, Carlsbad, CA, USA), adhering to the manufacturer’s standard protocol. The quality of RNA and the degree of purification were evaluated using a NanoDrop OneC instrument (Thermo Fisher Scientific, Waltham, MA, USA), examining the A260/A280 and A260/A230 wavelength ratios. These ratios should fall within the range of 1.8 to 2.2.

### 4.6. Preparation of RNA Libraries

RNA libraries were prepared utilizing the MGIEasy RNA Directional Library Prep Set (MGI, Shenzhen, China), in accordance with the manufacturer’s protocol. Sequencing was conducted on the DNBSEQ-G400 platform (MGI, Shenzhen, China) in paired-end reading mode, with a read length of 100 bp.

### 4.7. RNA Sequencing Data Processing

The quality of the obtained data was assessed utilizing the FastQC software (version 0.12.0). When necessary, adapter trimming and filtering of low-quality reads were conducted using Trimmomatic (version 0.33) [43]. Read mapping to the reference genome (GRCh38/hg38) was per-formed using Hisat2 software (version 2.2.1) [44]. The htseq-count program (version 2.2.1) was employed to enumerate reads for each transcript [45].

### 4.8. Identification of Differentially Expressed Genes (DEG)

Differential expression analysis was conducted utilizing the DESeq2 package (version 1.46.0) in R (version 4.2.0) [46]. Genes exhibiting a *p*-value less than 0.001 in the analysis were classified as differentially expressed genes. DEG with a log2FoldChange (log2FC) threshold exceeding 4 were categorized as upregulated, whereas those with log2FC below −4 were categorized as downregulated.

### 4.9. Functional and Enrichment Analysis of DEG Pathways

Gene Ontology (GO) pathway enrichment analyses were conducted to elucidate bio-logical processes, cellular components and molecular functions. The Bioconductor package “org.Hs.eg.db” (version 3.20.0) and the “clusterProfiler” package (version 4.14.4) [47] were utilized for GO pathway enrichment analyses.

### 4.10. Protein–Protein Interaction (PPI) Network Construction and Subnetwork Identification

Up- and down-regulated DEGs were employed to construct a PPI network using the Search Tool for the Retrieval of Interacting Genes/Proteins (STRING) database [48]. Additionally, the Molecular Complex Detection (MCODE) plugin (version 2.0.3) [49] in the Cytoscape software (version 3.10.3) [50] facilitated the analysis of densely connected clusters in networks based on specific criteria (degree cutoff = 2, node score cutoff = 0.2, K-core = 2, and max depth = 100). Subsequently, the subnetworks with the highest scores for up- and downregulated genes were selected. For further analysis of the subnetworks, Metascape (v3.5.20240901) [51] was employed. For each PPI subnetwork, the genes with the highest degree values—calculated using the CytoHubba plugin (version 0.1) [52] in the Cytoscape software—were designated as hub genes (i.e., genes that are closely connected within the module and significantly associated with biological function).

## Data Availability

The data presented in this study are available in the article and Supplementary Materials.

## Supplementary Materials

The following supporting information can be downloaded at: https://www.mdpi.com/article/10.3390/ijms26010124/s1.
